# Study on the Core-Shell Structure of Gas-Assisted Coaxial Electrospinning Fibers: Implications for Semiconductor Material Design

**DOI:** 10.3390/mi17010020

**Published:** 2025-12-24

**Authors:** Rongguang Zhang, Xuanzhi Zhang, Jianfeng Sun, Shize Huang, Xuan Zhang, Guohuai Lin, Xun Chen, Zhifeng Wang, Jiecai Long, Weiming Shu

**Affiliations:** 1 State Key Laboratory of Precision Electronic Manufacturing Technology and Equipment, Guangdong University of Technology, Guangzhou 510006, China; 2 School of Electromechnical Engineering, Guangdong University of Technology, Guangzhou 510006, China; 3 School of Electromechanical Engineering and Automation, Foshan University, Foshan 528011, China

**Keywords:** gas-assisted coaxial electrospinning (GACES), coaxial nanofiber, semiconductors material, controllable core-shell structure

## Abstract

Gas-assisted coaxial electrospinning (GACES), a simple and versatile technique for the large-scale fabrication of coaxial nanofiber membranes, possesses significant industrial potential across advanced manufacturing sectors including semiconductors—particularly for fabricating high-precision dielectric layers, high-uniformity encapsulation materials, and flexible semiconductor substrates requiring tailored core-shell architectures. However, there is still a lack of relevant studies on the effective regulation of the core-shell structures of coaxial fibers based on GACES, which greatly limits the batch preparation and wide application of coaxial fibers. Finite element simulation analysis of the flow field and development of the coaxial jet mechanics model with a gas-driven flow field—two key methodologies in this study—successfully uncovered the influence mechanism of gas-assisted flow fields on the core-shell structures of coaxial nanofibers. By adjusting the gas-assisted flow fields parameters, we reduced the total diameter of coaxial fibers by 47.33% (average fiber diameter: 334.12 ± 16.29 nm → 175.98 ± 1.18 nm), decreased the shell thickness by 72.98%, increased the core-shell ratio by 289% (core-shell ratio: 0.49 → 1.91), and improved the uniformity of the total diameter distribution of coaxial fibers by 30.64%. This study delivers a practical conceptual framework and robust experimental underpinnings for the scalable fabrication of coaxial nanofiber membranes with controllable core-shell structures, thereby promoting their practical application in semiconductor devices such as ultra-thin dielectric layers, precisely structured encapsulation materials, and high-uniformity templates for nanoscale circuit patterning.

## 1. Introduction

Coaxial nanofibers, with their tailored core-shell architecture, are pivotal for advancing nanoscale electronic devices (e.g., thin-film transistors, flexible integrated circuits) by enabling the precise regulation of carrier transport and interfacial stability [1,2,3,4,5,6,7,8,9,10,11]. However, the scalable fabrication of morphologically tunable coaxial nanofibers remains a bottleneck for industrial translation—conventional methods (template synthesis, self-assembly, traditional coaxial electrospinning) suffer from low productivity, complex processing, or poor structural uniformity [12,13,14,15,16,17,18,19].

Emerging hybrid techniques have addressed partial limitations: solution blow spinning (SBS) uses high-speed gas for high production rates but lacks an electric field—limiting morphology control [20,21,22,23]; electroblowing integrates electrostatic force and gas flow to balance productivity and uniformity but has rarely been applied to coaxial structures [24,25,26,27,28,29]. Notably, gas-assisted coaxial electrospinning (GACES)—a configuration combining electrostatic repulsion and directed compressed air jets—has emerged as a promising approach, yet systematic research on its ability to enable the large-scale fabrication of morphologically tunable coaxial nanofibers is scarce [30,31].

This paper investigates the influence of gas-assisted flow on the core-shell structure of coaxial nanofibers. Finite element simulation analysis of the flow field and development of the coaxial jet mechanics model with a gas-driven flow field—two key methodologies in this study—successfully uncovered the influence mechanism of gas-assisted flow fields on core-shell structures of coaxial nanofiber. Meanwhile, the quantitative analysis of the experimental results was carried out to realize the quantitative characterization of the changes of core/shell thickness, core/shell thickness uniformity, coaxial fibers’ average diameter, and distribution interval under different gas-assisted flow field parameters. Finally, experiments demonstrate that GACES reduces the average diameter and shell thickness of coaxial fibers by 47.33% and 72.98%, respectively, while narrowing the diameter distribution range by 30.64%. These advancements are expected to provide a practical conceptual framework and robust experimental underpinnings for the scalable fabrication of coaxial nanofiber membranes with controllable core-shell structures, thereby promoting their practical application in semiconductor device manufacturing—specifically in the fabrication of ultra-thin dielectric layers, high-uniformity encapsulation materials, and high-precision templates for nanoscale circuit patterning.

## 2. Materials and Methods

### 2.1. Material

Polyvinyl alcohol (PVA-220, hydrolysis degree: 87~89%, Kuraray, Tokyo, Japan), polyethylene oxide (PEO-N3000, M = 400,000 g·mol^−1^, DOW, Midland, MI, USA) and deionized water were obtained from Aladdin Chemical Co. (Riverside, CA, USA). All reagents were of analytical grade and used as received.

### 2.2. Fabrication of Nanofiber Membrane

**Figure 1:**
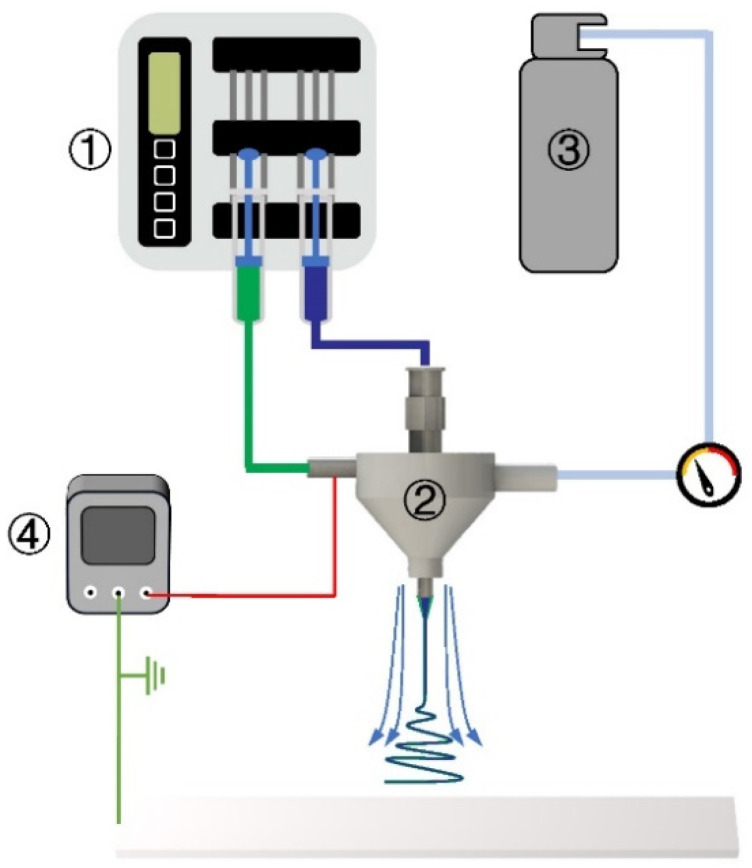
Schematic diagram of GACES: ① double-channel pump, ② gas-assisted coaxial electrospinning nozzle, ③ compressed gas, ④ high-voltage power supply.

The PVA particles and PEO powder were, respectively, added into the beaker filled with deionized water and then stirred and dissolved at 90 °C for 24 h to obtain a 2% PVA solution (core solution) and a 6.5% PEO solution (shell solution). A self-built experimental setup was employed (Figure 1). In this study, a coaxial gas duct nozzle (inner diameter: 2.78 mm) and a coaxial electrospinning needle (inner/outer diameters: 1.6/2.1 mm and 0.7/1.07 mm, respectively) were selected. The needle was charged to +15 kV via a power supply. The PEO shell solution and PVA core solution were pumped through the nozzle at 1 mL/h and 0.1 mL/h, respectively, using a dual-channel pump. The gas-assisted flow pressure was regulated to 0/5/10/15/20/25/30/40 kPa with a valve. The needle was positioned 25 cm above a grounded flat metal collection plate. Spinning was conducted at 25 °C and 20% RH. Polymer jets ejected from the needle tip were collected on the grounded plate, yielding ultra-fine coaxial nanofiber membranes.

### 2.3. Characterization and Deposition Evaluation of Nanofiber

The surface morphology of the nanofibers was observed by scanning electron microscopy (SEM, TESCAN MIRA3, Brno, Czech Republic). The distribution of nanofiber diameters was calculated from the SEM images using ImageJ software (NIH, Bethesda, MD, USA, version R1). In order to verify the core-shell structure of the nanofibers, coaxial nanofibers were deposited on a 300-mesh copper grid, and then TEM (HT7700, Hitachi, Tokyo, Japan) was utilized to observe the nanofibers at an acceleration voltage of 100 kV. A total of 100 nanofibers were randomly selected from SEM/TEM images of each nanofiber membrane sample for diameter and core-shell thickness measurements, and the diameter and core-shell thickness distributions of the coaxial nanofibers were analyzed and presented.

### 2.4. Numerical Simulation of Flow Field

ANSYS Fluent 2021 R1 software was used to construct the three-phase laminar transient model of a gas-assisted coaxial electrospinning for finite element simulation analysis. In order to obtain the velocity distribution of different phase fields under the same axis distance, test points were set up for different phase fields at the same axis distance.

## 3. Results and Discussion

### 3.1. Theoretical Research of Electrospinning Instability by Introducing Gas-Assisted Flow

#### 3.1.1. Simulation Analysis of Flow Field in Gas-Assisted Coaxial Electrospinning

By using ANSYS Fluent 2021 R1 software, the three-phase flow field (core/shell/gas-flow) model of gas-assisted coaxial electrospinning was established, and the three-phase flow field coupling simulation was carried out.

**Figure 2:**
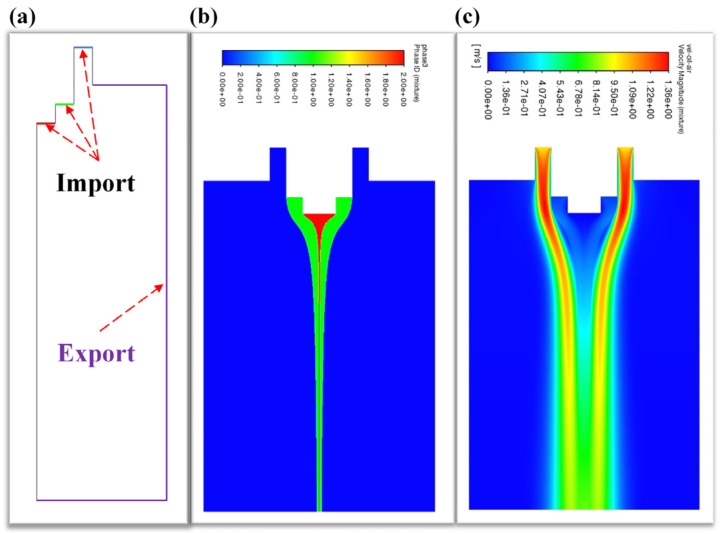
Diagram of the three-phase flow field (core/shell/gas-flow) model establishment and simulation results: (**a**) boundary condition settings of the three-phase flow import and export (red: core-flow import; green: shell-flow import; blue: gas-flow import; purple: export); (**b**) the three-phase field distribution; (**c**) velocity distribution cloud diagram.

In Figure 2a, the import and export boundary conditions for the three-phase flow field are set. According to the actual situation, the import quantities for the core/shell layer/gas flow field are set to 0.1/1/10 mL/h, respectively, at the red/green/blue boundaries in Figure 2a. The export conditions at the model boundary are set to 0 Pa, at the purple boundary export is shown in Figure 2a. By the three-phase flow field coupling simulation, the results of the phase field distribution and velocity distribution cloud diagram can be obtained, as shown in Figure 2b,c. From Figure 2b,c, we can see that the diameter of the core/shell phase gradually decreases along the negative direction of the Z-axis from the nozzle, obviously presenting the result that the core/shell phase is rapidly stretched by the gas-flow phase. Meanwhile, along the negative direction of the Z-axis, the overall flow rate of the core/shell field gradually increases.

**Figure 3:**
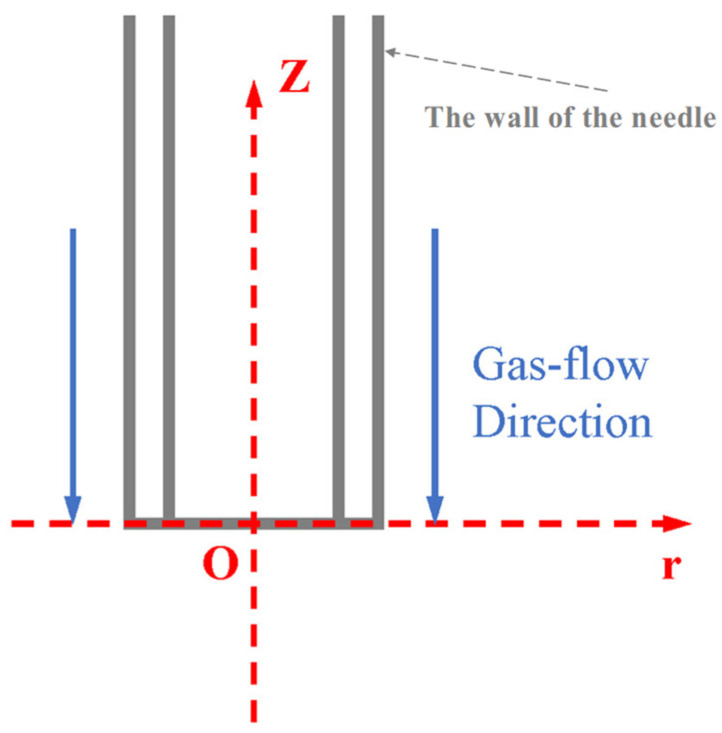
Coordinate system diagram of the gas flow at the needle.

As shown in Figure 3, the gas flow is sprayed from the +z direction to the zero point. To create the coordinate system, the center point of the needle outlet is the center point, and define +z as the positive directions of the velocity of the axial flow fields. Setting test points for the core/shell/gas-flow field, respectively, at 5/7.5/10/12.5/15/20/25/30/35/40/45 mm away from the nozzle outlet, we obtained the flow rate distribution curves of the three phase fields along the negative direction of the Z-axis from the nozzle, respectively. By analyzing the flow rate of the core/shell/gas-flow field under the same axis distance, we can obtain the variation in the flow rate ratio of the core/shell-flow field compared to along the negative direction of the Z-axis from the nozzle.

**Figure 4:**
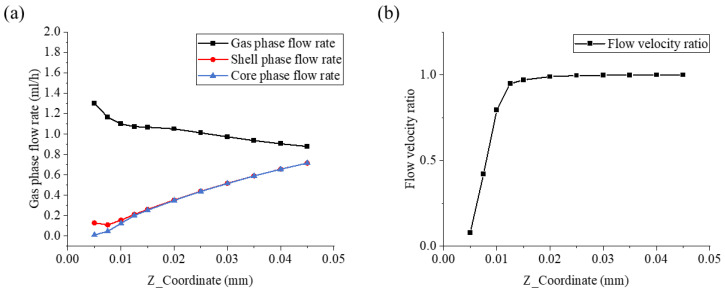
The velocity distribution along the negative direction of the Z-axis: (**a**) the velocity distribution of the core, shell and gas phase along the negative direction of the Z-axis; (**b**) the flow velocity ratio of the core/shell flow field.

From Figure 4a, it can be seen that the three-phase fluid is ejected from the nozzle outlet at their respective import velocities and moves forward along the negative Z-axis direction. During this process, the velocity of the gas flow field shows a continuous decaying trend. In sharp contrast, the velocities of the core/shell layer flow field start from relatively lower initial values and gradually increase in the same direction. This velocity change is essentially due to the viscous friction between different fluid phase interfaces. The high-speed moving gas flow will exert viscous drag forces on the adjacent core and shell layer fluids, transferring part of its kinetic energy to the originally slower core/shell layer fluids. As the kinetic energy is gradually transferred, the velocity of the gas flow field decreases due to energy loss, while the core and shell layer flow fields are driven by the drag force to accelerate. The difference in velocity between the three flow fields gradually narrows. This flow state adjustment is a dynamic equilibrium process with the kinetic energy of the gas flow field continuously transferring to the core/shell layer fluids until the velocities of the three-phase fluid tend to be consistent, ultimately forming a stable mixed flow state. This phenomenon is completely consistent with the actual flow characteristics observed in the experiment, clearly reflecting the basic laws of momentum transfer between multiphase fluids.

At the same time, we noticed that there was a certain gap in the flow rate of the core/shell-flow field in the initial very short period of time. Therefore, we described this process by using the flow rate ratio of the core/shell-flow field, as shown in Figure 4b. In Figure 4b, the flow ratio of the core/shell flow field gradually increases along the negative Z-axis direction from the nozzle and tends to a stable value. This indicates that the degree of difference in the influence of the gas flow field on the core/shell flow field gradually weakens and eventually reaches a stable state, marking that the multiphase flow system moves away from the nozzle along the negative Z-axis direction and enters a steady-state flow in momentum balance. The analysis of this phenomenon to some extent reveals the physical essence of the velocity changes of the core/shell/gas fluid flow, indicating the effective stretching effect of the gas layer on the coaxial jet, which is beneficial for the refinement of fibers and the improvement of the uniformity of fiber diameters.

#### 3.1.2. Mechanical Modeling and Analysis of Gas-Assisted Flow Coaxial Electrospinning Jet

Typically, the electrospinning process involves two characteristic stages: the initial stage features steady axial stretching of the jet driven by the combined effects of extrusion force, electrostatic force, viscous force, and gravity. As the jet morphology remains temporally invariant during this period, it is considered a linear stable motion phase. In this stage, the electrostatic force initially surpasses surface tension to draw the polymer solution out of the needle tip, gradually developing a Taylor cone morphology that characterizes the linear stretching stage. In the bending instability stage, the jet originating from the Taylor cone experiences whipping motion driven by charge repulsion forces, achieving rapid diameter reduction through non-axisymmetric stretching before solvent evaporation-induced solidification with the nanofiber randomly oriented on the collector.

In coaxial electrospinning technology, experiment parameters (flow rate ratio, voltage, solution viscosity, etc.) critically govern the core-shell nanofibers morphology. Central to this process is the stable Taylor cone—a dual-phase coaxial structure consisting of concentrically aligned inner and outer polymeric layers. Despite its composite structure, the cone maintains the Taylor cone shape, thereby enabling the formulation of the relevant theoretical model.

Meanwhile, the movement of the charged stable jet in the electric field in the initial stage of electrospinning can be regarded as a one-dimensional steady motion, so the one-dimensional steady fluid model can be used to analyze the behavior of the coaxial electrospinning process [32,33,34].

**Figure 5:**
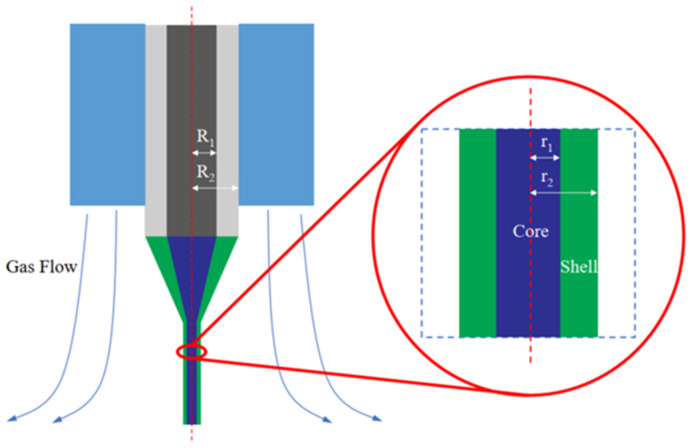
Schematic diagram of the core-shell jet movement of GACES.

We provide a schematic diagram of the gas-assisted coaxial electrospinning process, as shown in Figure 5.

For the subsequent model derivation, we first set the relevant parameters, as shown in Table 1.

According to the conservation of mass, we can obtain the following Equations (1) and (2).

(1) $$Q_{1}=\pi {r_{1}}^{2}(t)\rho_{1}u_{1}(t)$$

(2) $$Q_{2}=\pi [{r_{2}}^{2}\left(t\right)-{r_{1}}^{2}(t)]\rho_{2}u_{2}(t)$$

Therefore, by transforming Equations (1) and (2), respectively, we can obtain Equations (3) and (4).

(3) $$u_{1}\left(t\right)=\frac{Q_{1}}{\pi {r_{1}}^{2}(t)\rho_{1}}$$

(4) $$u_{2}\left(t\right)=\frac{Q_{2}}{\pi [{r_{2}}^{2}\left(t\right)-{r_{1}}^{2}(t)]\rho_{2}}$$

During the coaxial electrospinning process, the charged jet will have both surface current and volume current, so the total charge of the jet is composed of the surface charge and the volume charge.

According to the conservation of charge, we can obtain the following Equations (5)–(10).

(5) $$I_{1}=I_{11}+I_{12}$$

(6) $$I_{11}=2\pi r_{1}\left(t\right)\sigma_{1}u_{1}\left(t\right)$$

(7) $$I_{12}=k_{1}\pi {r_{1}}^{2}(t)E$$

(8) $$I_{2}=I_{21}+I_{22}$$

(9) $$I_{21}=2\pi r_{2}\left(t\right)\sigma_{2}u_{2}\left(t\right)$$

(10) $$I_{22}=k_{2}\pi [{r_{2}}^{2}\left(t\right)-{r_{1}}^{2}(t)]E$$

Therefore, by combining Equations (7) and (10), we obtain the relationship between the core jet radius and the total jet radius when only considering the influence of the electric field, as shown in Equation (11).

(11) $${r_{2}}^{2}\left(t\right)=\left(1+\frac{I_{22}}{I_{12}}\frac{k_{1}}{k_{2}}\right){r_{1}}^{2}\left(t\right)$$

Generally, when building a coaxial electrospinning model, we should consider the inertia, viscosity, statics, voltage, and surface tension of the fluid. It is worth noting that in the initial stage, the electric field force is much greater than other forces and becomes the dominant force; in the bending instability stage, it is mainly affected by the electric field force and viscoelastic force, and the resultant force between the two tends to zero; in the final stage, the acceleration of the jet motion tends to zero. However, this research scope here belongs to the initial stage, so we ignore other internal forces except the electric field force. Therefore, from Newton’s second law, we can construct Equations (12)–(17).

(12) $$\frac{du_{1}\left(t\right)}{dt}=\frac{F_{E1}+F_{s-c}}{m_{1}}$$

(13) $$m_{1}=\pi {r_{1}}^{2}\left(t\right)\rho_{1}u_{1}\left(t\right)$$

(14) $$F_{E1}=\sigma_{1}E$$

(15) $$\frac{du_{2}\left(t\right)}{dt}=\frac{F_{E2}+F_{c-s}+F_{g-s}}{m_{2}}$$

(16) $$m_{2}=\pi [{r_{2}}^{2}\left(t\right)-{r_{1}}^{2}\left(t\right)]\rho_{2}u_{2}\left(t\right)$$

(17) $$F_{E2}=\sigma_{2}E$$

At the same time, according to the law of internal friction, we obtain Equations (18)–(20).

(18) $$F_{s-c}=2\mu_{1}[u_{2}\left(t\right)-u_{1}\left(t\right)]$$

(19) $$F_{c-s}=-F_{s-c}$$

(20) $$F_{g-s}=2\mu_{3}\;u_{3}\left(t\right)$$

Finally, combining the above equation, we derived the differential equations of the total radius of the jet and core jet radius with respect to time, as shown in Equations (21) and (22).

(21) $$A_{11}\frac{dr_{1}\left(t\right)}{dt}+A_{12}r_{1}\left(t\right)+A_{13}{r_{1}}^{2}\left(t\right)=0,\;A_{11}=2,\;A_{12}=2\mu_{1}\left(\frac{Q_{2}\rho_{1}I_{12}k_{2}}{{Q_{1}}^{2}\rho_{2}I_{22}k_{1}}-\frac{1}{Q_{1}}\right),\;A_{13}=\frac{{\rho_{1}}^{2}I_{11}I_{12}}{2k_{1}{Q_{1}}^{3}}$$

(22) $$A_{21}\frac{dr_{2}\left(t\right)}{dt}+A_{22}r_{2}\left(t\right)+A_{23}(u_{3}\left(t\right)){r_{2}}^{2}\left(t\right)=0,\;A_{21}=2,\;A_{22}=2\mu_{1}\left(\frac{1}{Q_{2}}-\frac{Q_{1}\rho_{2}I_{12}k_{2}}{{Q_{2}}^{2}\rho_{1}I_{22}k_{1}}\right),\;A_{23}(u_{3}\left(t\right))=\frac{{\rho_{2}}^{2}I_{21}I_{22}}{2k_{2}{Q_{2}}^{2}}+\frac{2\pi \rho_{2}\mu_{3}}{{Q_{2}}^{2}}\frac{I_{22}k_{1}}{I_{12}k_{2}+I_{22}k_{1}}u_{3}\left(t\right)$$

Depending on whether gas-assisted flow is added, we solve the above differential equations of the total radius of the jet and core jet radius separately.

When $u_{3}\left(t\right)=0$, due to $r_{1}\left(0\right)=R_{1}$, from Equations (11) and (21), we obtain Equations (23) and (24).

(23) $$r_{1}\left(t\right)=R_{1}{\left[e^{\frac{A_{12}}{A_{11}}t}+(e^{\frac{A_{12}}{A_{11}}t}-1)\frac{R_{1}A_{13}}{A_{12}}\right]}^{-1}$$

(24) $$r_{2}\left(t\right)=R_{1}{\left[e^{\frac{A_{12}}{A_{11}}t}+(e^{\frac{A_{12}}{A_{11}}t}-1)\frac{R_{1}A_{13}}{A_{12}}\right]}^{-1}\sqrt{1+\frac{I_{22}}{I_{12}}\frac{k_{1}}{k_{2}}}$$

**Figure 6:**
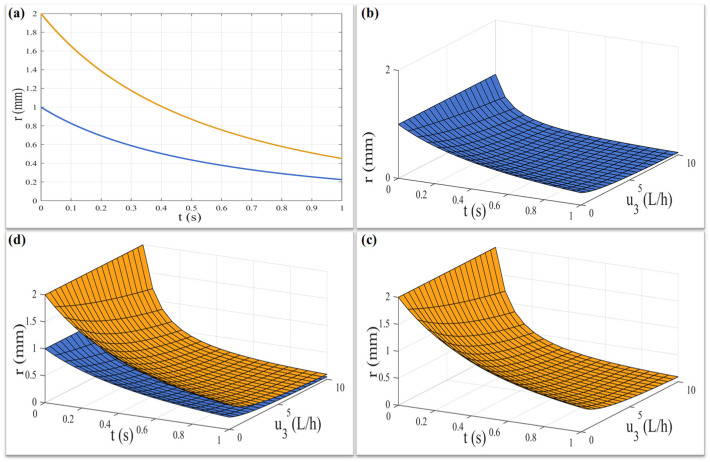
The trend diagram of the jet radius changing with time (core: blue, total: yellow): (**a**) only the influence of the electric field is considered; (**b**–**d**) the influence of the electric field and gas-assisted flow field are considered.

According to the above solutions of the differential equations of total radius of the jet and core jet radius given by Equations (23) and (24), we can obtain the trend graph of the total radius of the jet and core jet radius changing with time when $u_{3}\left(t\right)=0$, as shown in Figure 6a.

When $u_{3}\left(t\right)>0$, due to $r_{2}\left(0\right)=R_{2}$, from Equations (11) and (22), we obtain Equations (25) and (26).

(25) $$r_{2}\left(t\right)=R_{2}{\left[e^{\frac{A_{22}}{A_{21}}t}+(e^{\frac{A_{22}}{A_{21}}t}-1)\frac{R_{2}A_{23}\left(u_{3}\left(t\right)\right)}{A_{22}}\right]}^{-1}$$

(26) $$r_{1}\left(t\right)=R_{2}{\left[e^{\frac{A_{22}}{A_{21}}t}+(e^{\frac{A_{22}}{A_{21}}t}-1)\frac{R_{2}A_{23}\left(u_{3}\left(t\right)\right)}{A_{22}}\right]}^{-1}\sqrt{\frac{I_{12}k_{2}}{I_{12}k_{2}+I_{22}k_{1}}}$$

According to the above solutions of the differential equations of the total radius of the jet and core jet radius given by Equations (25) and (26), we can obtain the trend graph of the total radius of the jet and core jet radius changing with time when $u_{3}\left(t\right)>0$, as shown in Figure 6b–d.

Therefore, it can be seen from Figure 6a that for the coaxial jet affected only by the electric field force, the total jet radius and the core jet radius show a significant exponential decreasing trend with time, which is consistent with the actual situation of the experiment. Furthermore, as can be seen from Figure 6b–d, the coaxial jet, which was originally only affected by the electric field force, has its total jet radius and core jet radius reduced at a faster rate as the gas-flow field intervenes and its flow rate increases. This will be conducive to the rapid reduction in the fiber diameter within a limited time and improves the uniformity of the fiber diameter. Meanwhile, the gas-assisted flow field increases the motion constraints on the jet, which helps to collect the coaxial fibers in a specific area.

So far, the mechanical model for the single nozzle case of the gas-assisted coaxial electrospinning jet has been adequately described. When the mass production of multiple nozzles is really carried out, the related effects of the flow field and electric field between the nozzles must be considered in the subsequent work.

### 3.2. Experimental Verification

**Figure 7:**
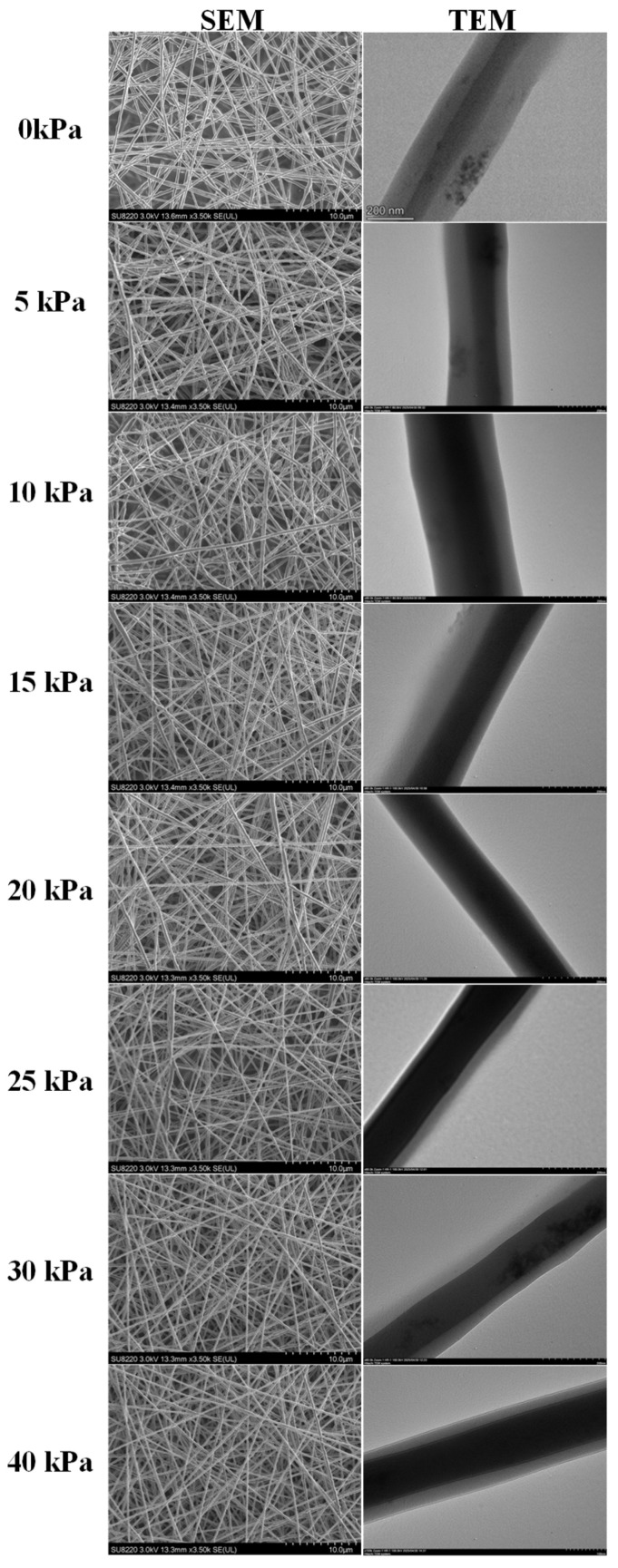
SEM and TEM characterization results of coaxial fiber under different gas-assisted pressures.

Firstly, by setting different gas-assisted pressure parameters, the SEM and TEM characterization results of the coaxial fiber membrane are shown in Figure 7.

As shown in Figure 7, when the gas-assisted pressure increased from 0 to 40 kPa, the coaxial fibers maintained a well-defined fiber morphology without bead-like or agglomerated structures. Furthermore, the fiber diameter decreased—this is because the gas flow enhanced the tensile force acting on the jet, facilitating fiber thinning.

Then, we statistically analyze the total diameter distribution of electrospinning coaxial fibers under the influence of the gas flow field. To quantify variations in total diameter distributions with higher accuracy, Gaussian fitting was applied to the total diameter data collected under different gas-assisted pressures. The fitting equation is as follows:

(27) $$y=y_{0}+\frac{A}{w\sqrt{\frac{pi}{2}}}e^{-2*{\left(\frac{x-x_{c}}{w}\right)}^{2}}$$

**Figure 8:**
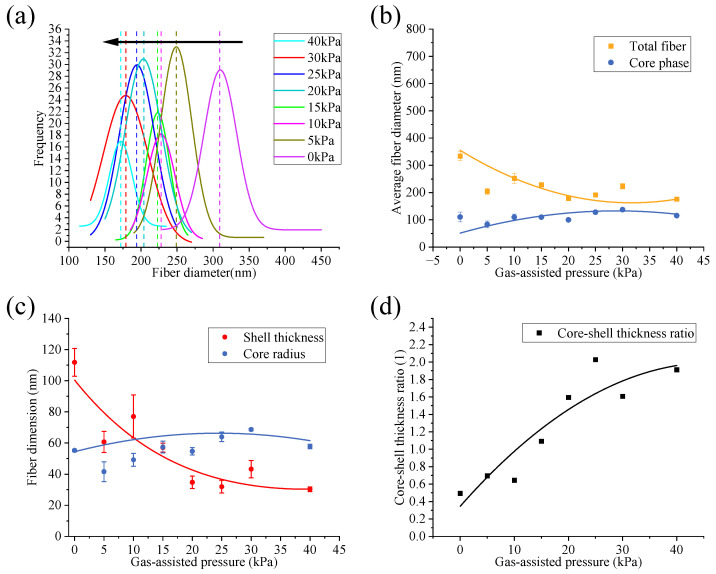
Quantitative analysis of coaxial fiber under different gas-assisted pressure: (**a**) fitting curve of coaxial fiber total diameter distribution; (**b**) the trend of the total diameter and the core phase diameter of the coaxial fiber; (**c**) the trend of core radius and shell thickness of the coaxial fiber; (**d**) core-shell thickness ratio of the coaxial fiber.

We then compared and analyzed the fitted curves, as shown in Figure 8a.

Meanwhile, the one-dimensional form of the standard Gaussian distribution is given by Equation (28).

(28) $$y=ae^{-\frac{{\left(x-b\right)}^{2}}{2c^{2}}}$$

where a denotes the peak height of the curve, b represents the peak center coordinates, and c corresponds to the standard deviation.

A comparison of Equations (27) and (28) reveals that a exhibits a positive correlation with $\frac{A}{w}$, while b and c are positively correlated with $x_{c}$ and w, respectively. As $y_{0}$ serves as an auxiliary fitting parameter, it will not be included in subsequent comparisons.

So, in Figure 8a, the center of the peak in the fitted curve (dotted line) of the total diameter frequency distribution histogram shows a leftward shifting trend (black arrow), indicating that the coaxial fibers are becoming thinner. At the same time, the standard deviation of the total diameter frequency distribution decreases from 22.65 nm (0 kPa) to 15.71 nm (40 kPa), indicating that the range of the total diameter has reduced by 30.64%, meaning the uniformity has improved. The experimental results have verified the inferences of the simulation and the mechanical model. The intervention and increase in the gas flow effectively refined the fiber diameter and enhanced the uniformity of the fiber diameter.

Furthermore, we conducted a statistical analysis on the total diameter and the core/shell thickness distribution of the coaxial fibers under different gas-assisted pressures, as shown in Figure 8b,c. In Figure 8b, it can be clearly observed that the total diameter exhibits a decreasing trend with increasing gas pressure. As the gas pressure increases (from 0 kPa to 40 kPa), the average value of the total diameter decreases from 334.12 ± 16.29 nm (0 kPa) to 175.98 ± 1.18 nm (40 kPa). Compared with the no-gas condition, the average value is reduced by a maximum of 47.33%. Figure 8c shows that the shell thickness follows a near-exponential decreasing trend with increasing gas pressure: in the initial stage, the shell thickness undergoes a sharp decrease as gas pressure rises with a maximum reduction of 72.98% relative to the no-gas condition. However, the core radius only exhibits slight fluctuations with increasing gas pressure, which varies between 24.68% and 24.31% of the no-gas value, and there is no significant change in the core radius. This phenomenon can be attributed to the coaxial jet stretching process: the introduction and gradual enhancement of the gas flow field accelerate the stretching and thinning of the coaxial jet. Since the force of the gas flow field acts first on the shell layer of the coaxial jet and then transfers to the core layer through the shell layer, the shell thickness shows a near-exponential decrease with the strengthened gas flow force. In contrast, the gas flow force has an insignificant effect on the core layer. For the core layer radius, this insignificant effect is mainly attributed to the mechanical coupling between the shell and the core being partial and indirect: the intervention of the gas flow field mainly changes the driving force acting on the jet, namely the air resistance and axial tensile force. These forces mainly act on the shell layer, which results in a very small effect of the gas flow field’s intervention on the core layer radius. Meanwhile, the intervention of the gas flow field accelerates the volatilization of the solvent in the shell fluid, which promotes the overall fiber curing and, to a certain extent, restricts the refinement effect of the core layer radius. As a result of this phenomenon, the core-shell ratio increases continuously with increasing gas pressure, specifically rising from 0.49 (0 kPa) to 1.91 (40 kPa), as shown in Figure 8d.

In summary, the introduction of an optimized coaxial auxiliary gas flow reduced the average total diameter by 47.33% while improving the uniformity by 30%. Meanwhile, without changing the diameter of the core, the thickness of the shell layer was controlled through gas-assisted pressures. This characteristic may be beneficial for drug-controlled release. All of the above experimental results are highly consistent with the conclusions derived from theoretical analysis.

## 4. Conclusions

In this study, we simulated the multiphase flow field during the GACES process via finite element analysis and established a jet mechanics model for this process. Through a series of formula derivations and experiments, we successfully revealed and verified the influence mechanism of the coaxial auxiliary gas flow on the diameter of coaxial fibers and the uniformity of their core-shell thickness. Finally, in the GACES experiments, the diameter of coaxial fibers with added gas flow assistance decreased by a maximum of 47.33% (from 334.12 nm to 175.98 nm). Meanwhile, compared with the no-gas condition, the shell thickness decreased by a maximum of 72.98% (from 111.82 nm to 30.26 nm), whereas the core radius only showed slight fluctuations. Thus, the above results demonstrate that the addition of a coaxial gas flow field can effectively promote the refinement of coaxial fibers and the regulation of shell thickness as well as improve the uniformity of coaxial fibers. These advancements are expected to deliver a practical conceptual framework and robust experimental underpinnings for the scalable fabrication of coaxial nanofiber membranes with controllable core-shell structures, thereby promoting their practical application in semiconductor devices such as ultra-thin dielectric layers, precisely structured encapsulation materials, and high-uniformity templates for nanoscale circuit patterning.

In future studies, we will focus on the large-scale fabrication of coaxial fibers via gas-assisted coaxial electrospinning. However, at least the following issues still need to be addressed: (1) developing a multi-nozzle module with uniform liquid and gas supply; (2) mitigating the impact of the gas flow environment and optimizing the structure of the multi-nozzle module; and (3) developing a quality evaluation system for coaxial nanofibers and related equipment for large-scale fabrication.

## Figures and Tables

**Table 1:** Equation parameter table.

Physical Symbols	Physical Meaning	Unit
$R_{1}$	Coaxial needle core radius	mm
$R_{2}$	Coaxial needle total radius	mm
$r_{1}(t)$	Core jet radius	mm
$r_{2}\left(t\right)$	Total radius of the jet	mm
$\left[r_{2}(t)-r_{1}(t)\right]$	Shell jet thickness	mm
$Q_{1}$	Core jet flow	kg/h
$Q_{2}$	Shell jet flow	kg/h
$Q_{3}$	Gas-assisted flow	kg/h
$\mu_{1}$	Dynamic viscosity of core jet flow	Pa·s
$\mu_{2}$	Dynamic viscosity of shell jet flow	Pa·s
$\mu_{3}$	Dynamic viscosity of gas-assisted flow	Pa·s
$u_{1}(t)$	Core jet flow rate	L/h
$u_{2}(t)$	Shell jet flow rate	L/h
$u_{3}(t)$	Gas-assisted flow rate	L/h
$\rho_{1}$	Core fluid density	kg/L
$\rho_{2}$	Shell fluid density	kg/L
I	The total charge of the jet	C
$I_{1}$	The charge of the core jet	C
$I_{2}$	The charge of the shell jet	C
$I_{11}$	The surface charge of the core jet	C
$I_{12}$	The volume charge of the core jet	C
$I_{21}$	The surface charge of the shell jet	C
$I_{22}$	The volume charge of the shell jet	C
$\sigma_{1}$	The surface charge density of the core jet	μC/m^2^
$\sigma_{2}$	The surface charge density of the shell jet	μC/m^2^
$k_{1}$	The conductivity of the core jet	S
$k_{2}$	The conductivity of the shell jet	S
E	Electric field strength	N/C
$F_{E1}$	The electric field force on the core jet	N
$F_{E2}$	The electric field force on the shell jet	N
$F_{s-c}$	The force of the shell fluid on the core fluid	N
$F_{c-s}$	The force of the core fluid on the shell fluid	N
$F_{g-s}$	The force of the gas flow fluid on the shell fluid	N
$A_{11},A_{12},A_{13},A_{21},A_{22},A_{23}(u_{3}\left(t\right))$	Coefficient term	/

## Data Availability

The data that support the findings of this research are available from the corresponding author upon reasonable request.
